# Supplementary material for: [68Ga]Ga-NODAGA-E[(cRGDyK)]2 and [64Cu]Cu-DOTATATE PET Predict Improvement in Ischemic Cardiomyopathy
Source: Diagnostics (Basel). 2023 Jan 11;13(2):268. doi: 10.3390/diagnostics13020268 (PMC9857952; doi:10.3390/diagnostics13020268)
Supplement: Supplementary file 1 [file diagnostics-13-00268-s001.zip › diagnostics-2098533-supplementary.pdf]

Supplementary

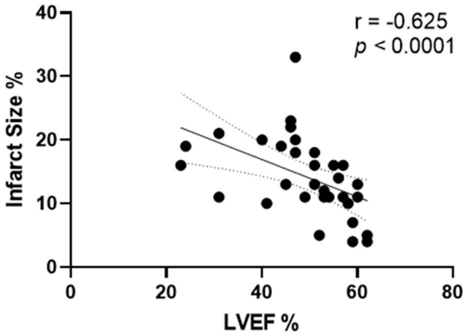

S1. Correlation between infarct area and LVEF.

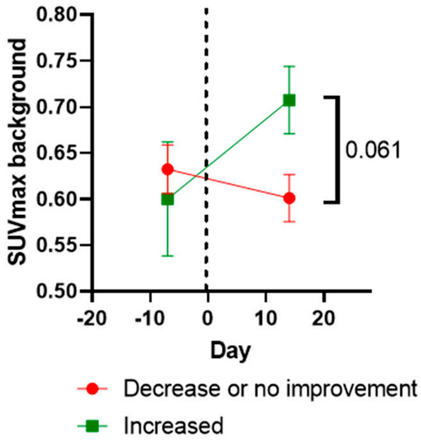

S2 [68Ga]Ga-RGD uptake in the remote area of the heart .

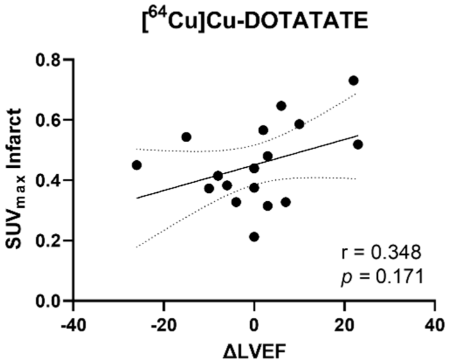

S3 [64Cu]Cu-DOTATATE uptake correlation with ΔLVEF.

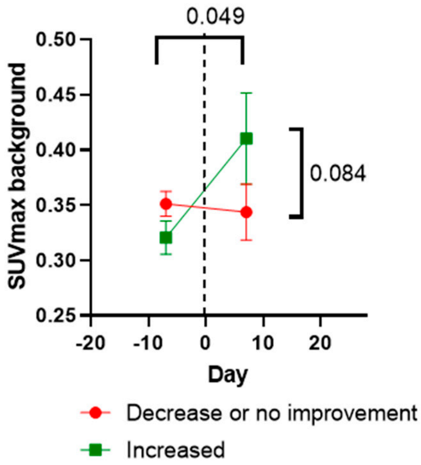

S4 [^{64}Cu]Cu-DOTATATE uptake in the remote area of the heart.

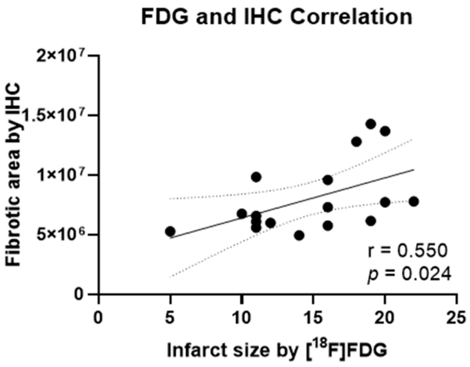

S5 Correlation between infarct size by 2-[^{18}F]FDG blackout and fibrotic area on immunohistochemistry (IHC) by Masson's Trichrome.
